# Knowledge Gaps in the Understanding of Antimicrobial Resistance in Canada

**DOI:** 10.3389/fpubh.2021.726484

**Published:** 2021-10-20

**Authors:** Kayley D. McCubbin, R. Michele Anholt, Ellen de Jong, Jennifer A. Ida, Diego B. Nóbrega, John P. Kastelic, John M. Conly, Matthias Götte, Tim A. McAllister, Karin Orsel, Ian Lewis, Leland Jackson, Graham Plastow, Hans-Joachim Wieden, Kathy McCoy, Myles Leslie, Joan L. Robinson, Lorian Hardcastle, Aidan Hollis, Nicholas J. Ashbolt, Sylvia Checkley, Gregory J. Tyrrell, André G. Buret, Elissa Rennert-May, Ellen Goddard, Simon J. G. Otto, Herman W. Barkema

**Affiliations:** ^1^Department of Production Animal Health, Faculty of Veterinary Medicine, University of Calgary, Calgary, AB, Canada; ^2^One Health at UCalgary, University of Calgary, Calgary, AB, Canada; ^3^O'Brien Institute of Public Health, University of Calgary, Calgary, AB, Canada; ^4^Snyder Institute for Chronic Diseases, University of Calgary, Calgary, AB, Canada; ^5^Department of Microbiology, Immunology and Infectious Diseases, Cumming School of Medicine, University of Calgary, Calgary, AB, Canada; ^6^Department of Pathology and Laboratory Medicine, Cumming School of Medicine, University of Calgary, Calgary, AB, Canada; ^7^Department of Medicine, Cumming School of Medicine, University of Calgary, Calgary, AB, Canada; ^8^Alberta Health Services, Calgary, AB, Canada; ^9^Department of Medical Microbiology and Immunology, Faculty of Medicine and Dentistry, University of Alberta, Edmonton, AB, Canada; ^10^Agriculture and Agri-Food Canada, Lethbridge Research Centre, Lethbridge, AB, Canada; ^11^Department of Biological Sciences, Faculty of Science, University of Calgary, Calgary, AB, Canada; ^12^Department of Agricultural, Food and Nutritional Science, Faculty of Agricultural, Life and Environmental Sciences, University of Alberta, Edmonton, AB, Canada; ^13^Department of Chemistry and Biochemistry, University of Lethbridge, Lethbridge, AB, Canada; ^14^Department of Physiology and Pharmacology, Cumming School of Medicine, University of Calgary, Calgary, AB, Canada; ^15^Department of Community Health Sciences, Cumming School of Medicine, University of Calgary, Calgary, AB, Canada; ^16^School of Public Policy, University of Calgary, Calgary, AB, Canada; ^17^Department of Pediatrics, Faculty of Medicine, University of Alberta, Edmonton, AB, Canada; ^18^Faculty of Law, University of Calgary, Calgary, AB, Canada; ^19^Department of Economics, Faculty of Arts, University of Calgary, Calgary, AB, Canada; ^20^Faculty of Science and Engineering, Southern Cross University, Lismore, NSW, Australia; ^21^Department of Ecosystem and Public Health, Faculty of Veterinary Medicine, University of Calgary, Calgary, AB, Canada; ^22^Alberta Precision Laboratories, Alberta Health Services, Calgary, AB, Canada; ^23^Department of Laboratory Medicine and Pathology, Faculty of Medicine and Dentistry, University of Alberta, Calgary, AB, Canada; ^24^Department of Resource Economics and Environmental Sociology, Faculty of Agriculture, Life and Environmental Science, University of Alberta, Edmonton, AB, Canada; ^25^HEAT-AMR Research Group, School of Public Health, University of Alberta, Edmonton, AB, Canada; ^26^Thematic Area Lead, Healthy Environments, Centre for Healthy Communities, School of Public Health, University of Alberta, Edmonton, AB, Canada

**Keywords:** antimicrobial resistance, One Health, antimicrobial stewardship, knowledge gaps, policy, Canada

## Abstract

Current limitations in the understanding and control of antimicrobial resistance (AMR) in Canada are described through a comprehensive review focusing on: (1) treatment optimization; (2) surveillance of antimicrobial use and AMR; and (3) prevention of transmission of AMR. Without addressing gaps in identified areas, sustained progress in AMR mitigation is unlikely. Expert opinions and perspectives contributed to prioritizing identified gaps. Using Canada as an example, this review emphasizes the importance and necessity of a One Health approach for understanding and mitigating AMR. Specifically, antimicrobial use in human, animal, crop, and environmental sectors cannot be regarded as independent; therefore, a One Health approach is needed in AMR research and understanding, current surveillance efforts, and policy. Discussions regarding addressing described knowledge gaps are separated into four categories: (1) further research; (2) increased capacity/resources; (3) increased prescriber/end-user knowledge; and (4) policy development/enforcement. This review highlights the research and increased capacity and resources to generate new knowledge and implement recommendations needed to address all identified gaps, including economic, social, and environmental considerations. More prescriber/end-user knowledge and policy development/enforcement are needed, but must be informed by realistic recommendations, with input from all relevant stakeholders. For most knowledge gaps, important next steps are uncertain. In conclusion, identified knowledge gaps underlined the need for AMR policy decisions to be considered in a One Health framework, while highlighting critical needs to achieve realistic and meaningful progress.

## Introduction

Since antimicrobial use (AMU) became widespread in healthcare, antimicrobial resistance (AMR) is increasing worldwide (1–3). Antimicrobials have saved hundreds of millions of human and animal lives and their discovery is a critical medical advance (1, 4). However, increasing AMR may vastly reduce future antimicrobial efficacy. The World Health Organization (WHO), the Food and Agriculture Organization of the United Nations (FAO), and the World Organization for Animal Health (OIE) agree that AMR is a serious threat to human and animal health and negatively impacts the environment (5). While the World Bank describes bacterial susceptibility to antimicrobials as a “public good” that needs global protection (1).

Due to a limited variety of efficacious antimicrobials, the same products or those within the same class are used in humans, animals, (6) agricultural crops, and aquaculture (7–9). This promotes bacterial resistance by increasing exposure of microbes to the same or similar antimicrobials (6), complicating AMR containment. Few drugs belonging to new antimicrobial classes have recently been released (3, 10–12) and efforts to reduce AMU and limit use of new antimicrobials reduce economic incentives for product development.

The extent of selection of resistant bacteria often reflects the degree of AMU, the type of antimicrobials used, and the effectiveness of infection prevention and control (13). The COVID-19 pandemic exacerbated the problem, as antimicrobial stewardship (AMS) efforts were often disregarded, with substantial AMU in COVID-19 patients for bacterial infections or prophylaxis (14–16). Furthermore, disinfectant use has dramatically increased, affecting the microbiome and potentially exacerbating AMR development (17, 18). Consequently, full impacts of the COVID-19 pandemic on development of AMR are unknown but may be substantial.

Current and projected AMR impacts require immediate and sustained action across human, animal, and environmental sectors using a true One Health approach, with multiple sectors communicating and collaborating to improve health outcomes while designing and implementing research, initiatives, policies, and legislation (19, 20). Livestock production is expected to be impacted, with a projected global decline of 2.6–7.5% annually (1). In addition to effects on animal health and welfare, consumer costs, food availability, and production system sustainability will be affected (21). It was estimated that by 2050, without interventions, every year there will be 10 million human deaths globally due to AMR infections (22). The incremental annual global healthcare costs due to AMR are expected to be ~US$0.33 to 1.2 trillion (1). In Canada, AMR cost our national healthcare system an estimated $1.4 billion in 2018 (21), with projected healthcare system costs reaching $120 billion by 2050 without interventions to slow predicted increases of bacterial resistance to first line antimicrobials from 26 to 40% (21). In addition to healthcare, the Canadian Council of Academies describes potential broader social impacts of AMR that include (21):

- decreases in social trust, social capital, quality of life, and equality among socio-demographic groups;
- weakened social connectivity;
- discrimination against those deemed at risk for or with AMR infections;
- unequal impacts of AMR, with higher risk for marginalized groups who experience poverty, homelessness, substance use disorder, overcrowding/poor housing/poor sanitation, and First Nations/Inuit/Metis populations); and
- a Canadian society that may become less open and trusting (i.e., less travel and increased support to close Canada's borders to immigrants and tourists).

There is substantial support regarding intricate connectivity of AMR in human, animal, and environmental sectors (6, 23–25), with spillover of AMR between microbial populations in livestock and humans (18). Furthermore, environmental bacteria are potential reservoirs for resistance genes acquired through exposure to antimicrobial residues from human, animal, and agricultural sources (23, 26–29). These environmental bacteria could transfer AMR traits to commensal and pathogenic bacteria. Transmission of AMR bacteria is influenced by trade, travel, and human and animal migration (3), making AMR a global issue that is not contained by political borders.

A One Health framework was used to identify three review areas to describe current knowledge gaps in Canada that need to be addressed before realistic, practice-oriented AMR mitigation strategies can be developed and implemented. These areas include: (1) treatment optimization; (2) surveillance of AMU and AMR; and (3) prevention of transmission of AMR. Herein, we have reviewed scientific literature and reports to identify the most pressing gaps in knowledge that currently hamper AMR prevention and control programs (summarized in Table 1). Where supporting data are lacking, expert opinion was used. Furthermore, requirements to address knowledge gaps are discussed, directing expert panels in identifying concrete next steps.

**Table 1:** Knowledge gaps that hamper prevention and control of antimicrobial resistance in Canada.

**Area**	**Knowledge gap**
Treatment optimization	- Extent of antimicrobial misuse in Canada - BMPs^a^ regarding antimicrobial prescribing in human/animal medicine - Economics of various efforts in lieu of AMU^b^ - Socio-economic/behavioral drivers of AMU (prescriber and patient perspectives) - Efficacy of widespread adoption of alternative therapies to AMU - Understand and shift perspectives that identify AMU is a harmless “cure all” - Identifying barriers and enablers of optimal human/animal AMU - BMPs to reduce livestock-associated prophylactic AMU - Rapid diagnostic testing to assist AMU decision-making
Surveillance	- Up-to-date prevalence estimates of AMR^c^ in the community, domestic animals, wildlife, production animals and the environment, not included in ongoing Canadian surveillance - Overall trends of AMR bacteria and their emergence in Canada - BMPs for integration of AMU/AMR data collection and reporting
Prevention of transmission of AMR	- Long-term efficacy of AMR mitigation efforts - BMPs for prevention of hospital-acquired AMR infectons - BMPs for reducing AMR in wastewater and subsequent impacts on human/animal health - How to reduce AMR prevalence in various resistance reservoirs - How to limit the risk of AMR in food systems - How to prevent cross-species AMR transmission - Quantitative risks associated with various incursion pathiways of AMR transmission *Development of AMR* - The direct relationship between AMU and AMR development - Role of the microbiome - Impact of heavy metals, cleaning agents and biocides, and other xenobiotic compounds on AMR development - How AMU in one health sector directly impacts AMR development in another sector (i.e., human AMU and animal AMR, and the reverse) - Relative importance of various routes of antimicrobial administration in AMR development - How to employ policy to effective limit AMR development *Role of the Environment* - Impact of human AMU/AMR on the environment - Impact of AMU/AMR in livestock industries on the environment - Relative importance of various environmental transmission routes (including transmission through ground water and livestock derived manure spreading, etc.) - Impact of antimicrobial residues in soil, water, and pastures - Economic impacts of reducing environmental AMR reservoirs and antimicrobial residues *Role of Wildlife* - Impact of AMR on wildlife health - Role of wildlife in transmission of AMR - Economic benefits of reducing AMR transmission from wildlife to livestock or humans

a *BMPs, Best management practices*.

b *AMU, Antimicrobial use*.

c *AMR, Antimicrobial resistance*.

## Treatment Optimization

AMU is considered one of the most important factors in development and spread of AMR, with misuse and overuse of particular concern (3, 18, 30). Examples of the latter by prescribers, patients or antimicrobial administrators (i.e., farmers) include: excessive use for disease prevention or treatment in lieu of good hygiene, inappropriate off-label use, treatment of non-bacterial illnesses, growth promotion in livestock, improper dosing (quantity, interval or duration), patient/administrator non-compliance, fraudulent formulation and incorrect antimicrobial selection (18, 30–33). To maintain the efficacy of available antimicrobial treatments, treatment optimization is crucial and includes, as a minimum:

- use of the least broad-spectrum antimicrobial for the infection;
- avoiding unnecessary prophylactic and broad-spectrum AMU;
- avoiding antimicrobial prescribing before bacterial culture and sensitivity;
- optimal dosing (i.e., quantity, interval, duration); and
- patient compliance.

Development of AMS best practices does not ensure their uptake by various stakeholders, for societal, cultural (34) and economic factors. For example, there is frequently prophylactic or empirical AMU in lieu of more costly solutions (i.e., diagnostics and/or focused treatment). Understanding AMU economics is vital to promote support of AMS and AMU reduction efforts, at policy, prescriber, and patient-levels, but currently lacking (Table 1). Specific societal and cultural factors in human medicine include-short term benefits, such as positive clinical outcomes and avoidance of clinical risks, maintaining prescriber-patient relationships, societal pressures and prescriber expectation outweighing long-term AMR community risks, and thus hampering judicious AMU (34). Antimicrobial prescribing can also be influenced by social hierarchies in the both the human (16) and veterinary medical settings (35). Even when AMS strategies in human hospitals were developed, they often did not include who should act and failed to account for multi-professional care teams and details on when to start/stop antimicrobials (36). Livestock-focused research suggests when AMR mitigation research is interdisciplinary, behavioral feasibility is also considered by identifying all actors in livestock AMU and power relationships (37).

To increase social science inclusion in AMR research and policy, focus groups with physicians, veterinarians, agricultural crop managers, livestock producers, and other relevant stakeholders should be utilized to identify factors hampering behavioral change and then to develop AMS interventions that ensure uptake. Integration with social science domains could identify considerations essential to each sector for support of AMU reduction strategies, in addition to described economic considerations. Close collaboration with social scientists is needed to successfully implement AMS programs.

Stewardship efforts can be supported by further development and availability of rapid diagnostic technology, to decrease empirical prescribing and increase appropriate antimicrobial treatment response times (16). An impediment to optimal dosing is the knowledge gap regarding quantitative relationships between AMU and AMR development, and interactions of resistant bacteria at the human, animal, and environmental interface (Table 1). Further research regarding a dose-response relationship of AMU and subsequent AMR development, including impacts and relative importance of number of antimicrobial doses, or duration of AMU in various contexts, is required to inform model development to understand impacts of AMU and mitigation efforts.

A key factor in reducing AMU is reducing disease prevalence/burden. Non-antimicrobial alternatives for infection prevention, e.g., vaccines and alternative therapies (i.e., phages, lysins, antimicrobial adjuvants, probiotics, and microbiome alterations) are being explored to prevent or treat infections (21). These alternatives should be considered crucial for treatment optimization, to reduce unnecessary AMU; however, their short and long-term efficacy is currently unknown (Table 1), and they have yet to dramatically reduce AMU.

### AMU in Humans

#### Current Knowledge

AMU for human health is increasing worldwide as reported in 2015 (3, 38), particularly in low- and middle-income countries (38). In Canada, as reported in the 2020 CARSS update (Canadian Antimicrobial Resistance Surveillance System) there was an increase in annual antimicrobial consumption, between 2014 and 2018, with a 28.6% increase in antimicrobial purchasing by hospitals, despite a 1.3% decrease in retail dispensing (39). Concurrently, there was a 10% increase in use of antimicrobials that should be reserved for suspected or confirmed multidrug-resistant infections, some of which are increasing (39). For example, the proportion of MDR invasive *Streptococcus pneumoniae* infections increased by 26% from 2013 to 2017 (39).

In 2018, 89.8% of antibiotics for human use in Canada were prescribed in community health care settings (e.g., by family physicians, dentists, pharmacists, nurse practitioners, etc.) and only 10.2% used in hospitals (39). In Canada, 30% of antimicrobial prescriptions dispensed through pharmacies (40) and 57% in long-term care facilities were unnecessary (41), highlighting opportunities to improve prescribing practices and patient expectations.

#### What Is Missing?

Most unnecessary AMU in humans is not related to gaps in prescriber knowledge, but instead to other factors at the provider and/or patient-level, interplaying with various contextual factors (42). Further understanding of these contextual prescribing factors represents a major knowledge gap that must be addressed for practical AMS recommendations to be developed and upheld (Table 1). For example, there is a perception of antimicrobials as “magic bullets” or a harmless “cure-all” (43). These perceptions must be altered to generate effective change in prescribing practices in all sectors and to prevent the addition of antimicrobials to medical regimes “just in case.”

There is limited public knowledge regarding the harms of inappropriate AMU and AMR implications (44). Receiving an antimicrobial prescription is part of the social contract of a medical appointment in human and veterinary medicine. Before substantial progress can be made, this gap in public knowledge regarding when AMU is appropriate must be addressed and prescribing guidelines improved to shift the dialogue and alter patient expectations. For example, “Using Antibiotics Wisely” is a national campaign developed by Choosing Wisely Canada to facilitate patient-physician conversations regarding unnecessary AMU in Canada (45). “Bugs and Drugs” is another resource developed by Alberta Health Services (AHS), and “Do Bugs Need Drugs” was developed by AHS and the British Columbia Centre for Disease Control (46, 47). Furthermore, an innovative University of Calgary AMS “app” has been modeled by many sites globally (48). These initiatives represent advances in treatment optimization and information availability. However, there is opportunity for substantial progress regarding uptake of prescribing recommendations, including sustained behavior change, increasing public knowledge and expectations, as well as altering the social environment, culture and value systems surrounding AMU.

### AMU in Animals

#### Current Knowledge

Extensive AMU for treatment and prevention of infectious diseases in livestock has supported development of current animal production systems (49). In 2016, the total volume of antimicrobials (excluding ionophores and chemical coccidiostats) to treat Canadian livestock was nearly four times the amount used in humans, with almost all used in production animals (50). However, the context of AMU must be considered, including the population correction unit (PCU) that enables standardization of antimicrobial product weight (mg) per unit of animal or human biomass (kg) (50). When the Canadian-specific animal PCU is considered, animal-intended antimicrobial distribution was only 1.3 times that prescribed for humans (50). Although the quantity of antimicrobials dispensed is not perfectly correlated to use, it provides a proxy to assess trends. The quantity of animal-intended antimicrobials dispensed in 2017 in Canada was 11% lower than in 2016; however, there was a 6% increase from 2017 to 2018 (39). In 2018 the animal sector represented 79% of AMU, the human sector was responsible for 21%, and crop AMU represented <1% (39). However, there were ~21 farmed animals for every human (51). Furthermore, antimicrobials intended for growth promotion [i.e., treatment/prevention of subclinical disease to improve health and increase production (52)] in broiler and turkey flocks decreased to zero between 2014 and 2018 (39).

As of December 1, 2018, all “medically important antimicrobials” (53) for veterinary use became limited to prescription-only access in Canada (33), which will improve assessment of AMU in Canada and reduce inappropriate use. Many antimicrobials deemed as “last resort” to treat infections in people are already restricted from use in livestock, or limited to prescription-only access, and livestock industries are adopting voluntary policies to avoid these compounds for disease prophylaxis, as exemplified by the Chicken Farmers of Canada AMU reduction strategy (54). Additionally, the Québec government restricted Category I AMU in food animals starting February 25th, 2019 (Category I antimicrobials are of high importance in human clinical disease) (55). Specifically, preventative use of Category I antimicrobials is banned, and clinical use is only permitted in livestock for cases where antimicrobials of a lower class of importance to human medicine will not be effective (e.g., based on culture and sensitivity) (55). Long-term impacts of this provincial stewardship program are unknown.

There are opportunities for further AMU reduction in Canadian livestock. For example, the majority of AMU on Canadian dairy farms is for mastitis treatment and prevention (56, 57). Blanket dry cow therapy practices are most common; at the end of lactation (start of the dry period), dairy cows are prophylactically treated with intramammary antibiotics to cure current bacterial infections and prevent new ones (58). Alternatively, selective dry cow therapy targets cattle expected to benefit from antibiotics (58), with no effect on animal production and udder health if cattle are selected appropriately (59–61). Therefore, this can reduce livestock-associated AMU in Canada.

This example highlights the importance of context when evaluating AMS initiatives. Research is required to develop best management practices to reduce livestock-associated AMU but maintain animal health and production (Table 1). In addition, research is underway on various approaches to reduce AMU in livestock, including vaccinations, pre- and probiotics, selection of animals less susceptible to disease (62–64), etc. However, their development and uptake has yet to dramatically reduce on farm AMU, with further work required.

Antimicrobials for companion animals accounted for only 1% of total antimicrobial sales in 2016 (50). However, companion animals are more likely to receive Category I and II antimicrobials (39) closely related to human medications. This, and the close proximity of humans and their pets creates potential for transmission of organisms resistant to highly important antimicrobials. AMR bacteria have been reported in companion animals. For example, methicillin-resistant *Staphylococcus pseudintermedius* (MRSP), causes common and sometimes untreatable skin and surgical site infections in dogs (65). Furthermore, humans with methicillin-resistant Staphylococcus aureus (MRSA), a bacteria of public health concern, can infect companion animals, which are a source of infection or reinfection (66).

#### What Is Missing?

Similar to human medicine, prescribing guidelines on appropriate companion animal and livestock AMU require improvement. However, to improve uptake of AMS recommendations, veterinarian-public expectations must also be altered to shift prescribing expectations and limit social pressure on prescribers. The Canadian Veterinary Medical Association provides AMU guidelines to improve veterinary prescribing decisions (67). Furthermore, relationships between companion animal AMR development or acquisition and impact on other species (including humans) or the environment are currently unknown (Table 1).

Current AMR impacts on wildlife and their contributions to dissemination of resistant bacteria or genes are unknown (18). Whereas AMR is reportedly higher in farmed animals vs. wildlife (68), AMR bacteria were reported in remote wildlife (69–71), questioning impacts of human activities on wildlife populations. Overall, the intricacies of microbial population interactions among various animal species and their relationship to human and environmental AMR and use are unknown.

### AMU in the Environment

#### Current Knowledge

Antimicrobials are used in agriculture for crop management and released into marine environments through aquaculture via feed or water (6–9, 39). Less than 1% of Canadian AMU is attributed to crop management (39), including streptomycin for treatment of fire blight (72). However, due to wastewater discharges (73–75), application of sewage-derived biosolids (76), and farm manure and animal production facility runoff (77), the environment is where human and animal AMU intersect, in addition to specific AMU for agricultural purposes (18). The environment is also a primary source of resistance genes (78) and a site for persistence and amplification (i.e., horizontal gene transfer) to pathogens of potential concern (79). Therefore, reducing human and animal AMU will not eliminate AMR (80).

Environmental reservoirs may facilitate maintenance of high concentrations of AMR bacteria due to on-going use of co-selecting agents, e.g., widespread use of biocides (18, 81) and disinfectants in municipal water and wastewater treatment (82, 83). *In vitro* studies demonstrated that using common herbicides on crops can modulate AMR to common antimicrobials in indicator bacteria (*Escherichia coli*) and foodborne pathogens (*Salmonella* spp.) (84). Additionally, in paleontological studies of soil cores, heavy metal pollution in historical industrial areas may have co-selected for AMR genes to antimicrobials of importance to human medicine before the advent of penicillin (85). There is evidence for global dissemination of *E. coli* that is highly resistant to wastewater treatment and has resistance genes to antimicrobials important to human medicine, with genetic similarity to virulence genes of urinary pathogenic *E. coli*, raising questions about this exposure pathway for humans (86, 87). The spread of resistant strains and resistance genes may be a dominant contributor to AMR maintenance, with sanitation and water treatment having a large role in reducing AMR transmission (88, 89).

#### What Is Missing?

Direct impacts of human or animal AMU or antimicrobial residues in the environment and impact of AMR in the environment and subsequent impacts on humans and animals are not well-characterized (Table 1). It is difficult to quantify impacts of human and animal AMU and AMR on the environment, but there is a strong correlation between socio-economic, health and environmental factors and AMR gene abundance in untreated human sewage (90).

AMU is a major risk factor for development of AMR bacteria and their environmental presence. Although broad exposure may occur via pathways such as drinking water, there are limited data to quantify direct human health effects from environmental AMR, another major knowledge gap (Table 1) (18, 91). Regardless, substantial reduction of antimicrobial misuse through treatment optimization/AMS programs can lessen selection pressure on microbial communities (1). AMS programs should also include reductions in direct application of manures/biosolids to land (92). Although the environment may have a quantitatively minor role compared to human-to-human and animal/food-to-human pathways, it may still be critical in overall AMR impact reduction (93), as demonstrated in developing areas (88). Therefore, environmental reservoirs should be integral to development of strategic AMR mitigation. First, there must be research to quantify AMR presence in the environment, impact of AMU in humans and animals, and other factors promoting AMR development and maintenance in the environment.

## Surveillance

### Current Knowledge

Surveillance is essential to demonstrate trends and monitor emerging and re-emerging AMR pathogens and AMU across all health sectors and provide data to support stewardship to address AMR (30). Surveillance data provide crucial information to identify areas for strategic interventions, increase understanding of the magnitude of AMR impacts and provide context to assess impacts of AMS interventions. Without sustained surveillance regarding AMR/AMU across the One Health continuum, public health authorities and policymakers in government and industry will lack information to craft and evaluate appropriate policy responses (30).

Surveillance for AMU/AMR in Canada has increased, but not all sectors or species affected by AMR are in a holistic system, which remains a compilation of multiple programs (94). There are currently surveillance systems at various levels of government aimed at data collection on AMR and AMU in Canadian settings such as hospitals, communities, and farms (30, 94). Ongoing National Canadian surveillance programs include (8, 30, 39, 94–98):

- Canadian Antimicrobial Resistance Surveillance System (CARSS): Incorporates epidemiological and laboratory AMR/AMU data from the Public Health Agency of Canada's surveillance systems (listed immediately below), from human, production animal, and food sources.
- Canadian Integrated Program for Antimicrobial Resistance Surveillance (CIPARS): Monitors trends in AMR/AMU for select bacteria mainly from humans, animals, and the food supply chain.
- Canadian Nosocomial Infection Surveillance Program (CNISP): Collects information on AMR/AMU for nosocomial infections in hospitalized human patients.
- Canadian Tuberculosis Laboratory Surveillance System: All tuberculosis cases diagnosed in Canada are reported (with/without treatment started) for: citizens, permanent residents, refugees, refugee claimants, and protected people. For temporary residents, only cases where treatment was started in Canada are reported.
- The Gonococcal Antimicrobial Surveillance Program (GASP-Canada): Laboratory surveillance data for Neisseria gonorrhoeae isolated by provincial microbiology laboratories to the National Microbiology Laboratory (NML).
- Pest Management Regulatory Agency (of Health Canada): Provides AMU data for crop production to CIPARS.
- The National Laboratory Surveillance of Invasive Streptococcal Disease (eSTREP): Passive and voluntary collaboration with provincial public health laboratories for *S. pneumoniae* and *Streptococcus pyogenes* surveillance, with some provinces only submitting a subset of isolates.

### What Is Missing?

Regrettably, these systems do not encompass the full scope of Canadian AMR and AMU. Data on environmental AMR are typically outside their mandates and therefore missing, and information is frequently not linked across systems (94). CIPARS is the only program purposefully designed to be an integrated AMR/AMU surveillance program, whereas others use various infectious disease platforms to collect AMR/AMU data (94). There are important gaps for a truly comprehensive, integrated, One Health AMR/AMU surveillance system in Canada (Table 1). Several of these gaps are reflected in the “Federal action plan on antimicrobial resistance and use in Canada” (99) and the “Pan-Canadian framework for action” (30) but they still exist.

**Figure 1:**
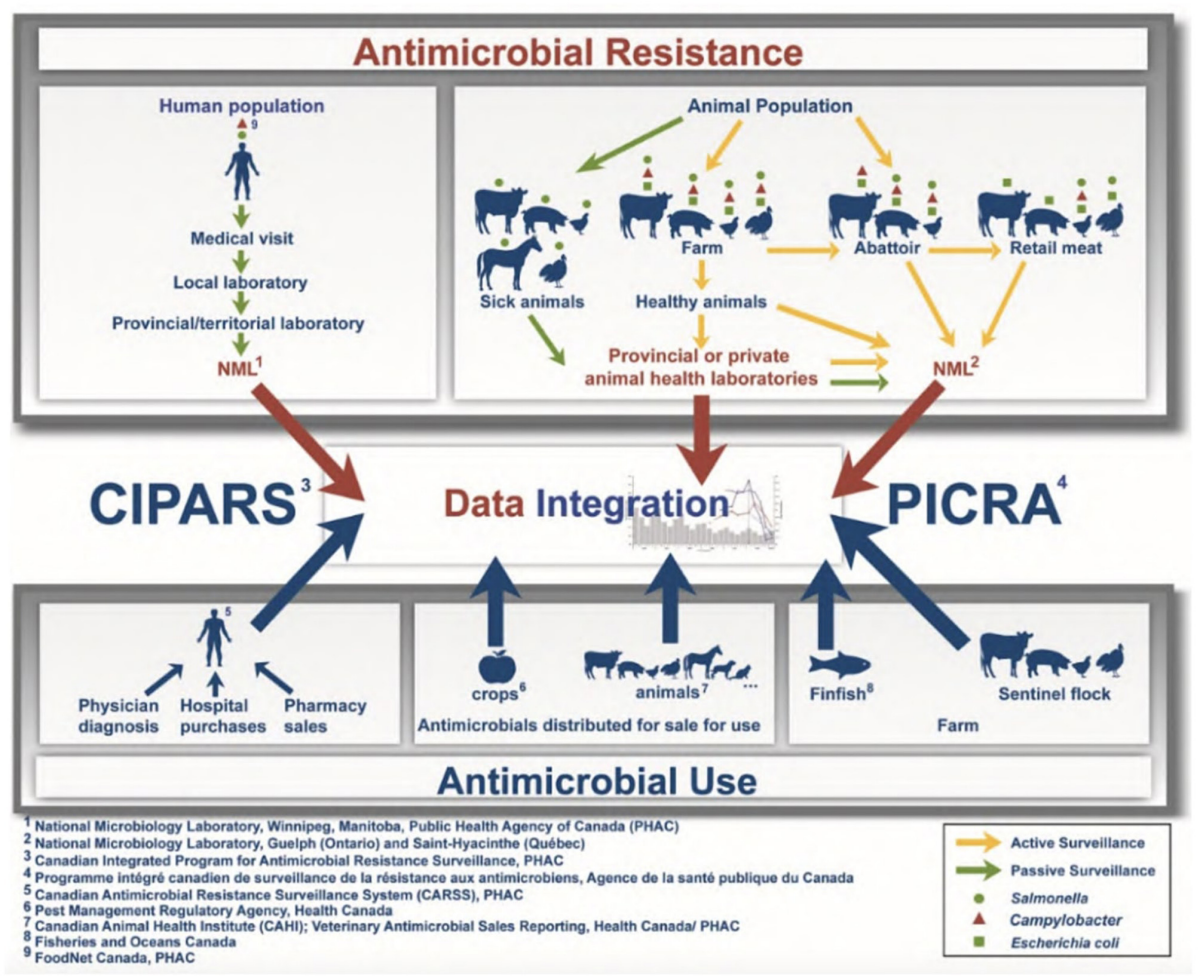
Canadian Integrated Program for Antimicrobial Resistance Surveillance program methods regarding AMR and AMU surveillance, from the Public Health Agency of Canada (51).

Without full integration and comparability of collected data, understanding is limited regarding local drivers of AMR and ensuing impacts. For example, CIPARS represents a national surveillance system with a One Health approach but consists only of active AMR surveillance for select bacteria in chickens, turkeys, pigs, dairy and recently, feedlot cattle, and passive reporting in humans and other animal species (Figure 1) (51). Further, on-farm components of CIPARS rely on a limited number of sentinel farms within their chicken, turkey, pig and feedlot programs, and some (e.g., pigs) are limited to final production phases. There is also a lack of information on AMR prevalence in companion animals and risks due to close proximity to humans.

Up-to-date AMR prevalence estimates for other species/sectors, e.g., prevalence in the general human population, on-farm data for multiple livestock species, and wildlife are lacking. This represents an urgent knowledge gap (Table 1); without current AMR prevalence estimates across all relevant sectors in Canada, there are no benchmarks to evaluate established mitigation strategies or identify important areas for policy development. Furthermore, comprehensive surveillance of AMR prevalence could inform patient treatments (3).

According to the Council of Canadian Academies, weaknesses in current Canadian AMR/AMU surveillance include limited data on: (1) infections of priority pathogens in community settings (i.e., non-hospitalized patients); (2) AMU in many regions of Canada; (3) AMR in pathogens of domestic animals and wildlife; (4) lack of an established federal/provincial/territorial surveillance system; and (5) lack of access to collected data (21). Furthermore, sustained surveillance of AMR development, persistence and transmission through the environment is not established. There are very limited data on resistance determinants in the environment. CIPARS does track AMU in some agricultural components (51), but there is no clear understanding of environmental AMR in Canada. CIPARS represents a substantial contribution to the surveillance and understanding of AMR in Canada and provides a world-recognized framework for integrated surveillance. It highlights the importance of continued AMR mitigation efforts (51), yet also identifies important surveillance gaps needing more coordination and resources.

To fully utilize and integrate data generated by CARSS and other programs and agencies globally, international standards for AMR and AMU data collection and reporting among human health, veterinary, and agricultural sectors are required, but absent (3, 94). Without comparable data among species, sectors, and regions, global understanding of AMU/AMR is impossible. In addition to implementation of reporting standards, data must be of good quality, and accessible to researchers and policymakers. There is also a need for measurable goals and evaluation criteria when considering surveillance data. Without practical and actionable goals that meet provincial and national needs, progress to control development of AMR cannot be measured or made (3).

Human health care is primarily provincially funded and regulated in Canada, whereas veterinary and environmental sectors have substantial federal and provincial/territorial components, requiring cooperation/collaboration among governments and engagement of all sectors and relevant stakeholders. In summary, due to the interconnectedness of AMR development, Canada could benefit from integration and standardization of human, animal, and environmental AMU/AMR data collection. This requires coordination of existing surveillance systems and addition of broader environmental considerations to capture local drivers of AMR.

## Prevention of Transmission of AMR

### Current Knowledge

Treatment optimization and AMS are important in preventing AMR transmission, as they reduce the overall burden of resistance on the microbiome, limiting risk of transmission. AMS efforts in human hospitals have improved AMU quality (100), and there are associations between reductions in livestock AMU production system AMR prevalence (6, 101). Additionally, there were decreases in human occupation-associated AMR infections in affected production systems (6). Therefore, it is very important to promoting reductions in unnecessary AMU across sectors.

Antimicrobials are important to treat, prevent, and control infectious diseases and maintain animal health and welfare in intensive livestock production (18, 49). By decreasing the reliance on AMU in intensive farming systems, emphasis on other forms of biosecurity must be increased, as well as farm infrastructure, management and breeding practices designed to improve animal health and resilience to reduce disease transmission risk, with increasing costs for producers and consumers.

Additionally, infection prevention and control procedures are invaluable to prevent transmission of AMR bacteria. Improved adjacent infection control efforts could further limit AMU. While this refers to sterility and hygienic practices for humans and animals (30), it also refers to maintaining healthy microbial populations that resist recolonization and opportunistic infections with resistant bacteria. Maintaining healthy microbial populations is critical for immunocompromised individuals, livestock, and crop production systems.

“*Every infection prevented is one that needs no treatment.”*

World Health Organization, 2015

### What Is Missing?

Despite new knowledge regarding the role of the environment in preventing AMR, much remains unknown; however, the environment is likely an important reservoir of resistance genes (2, 23, 27–29, 85). AMR development in the environment is attributed to AMU, plus other pharmaceutical agents and heavy metals (17, 84, 85, 102, 103). Data and knowledge required to undertake a human health risk assessment for environmental development and transfer and AMR include: (1) surveillance of clinical and environmental AMU, AMR, and their determinants; (2) epidemiological investigations of AMR outbreaks and sporadic cases; (3) identification of selection pressures in various environments and transmission to human-relevant bacteria; (4) links between AMU and resistance (human, laboratory, and/or field animal/crop); (5) AMR characteristics and their determinants; (6) links among AMR, virulence, and ecological fitness; (7) environmental fate of antimicrobial residues in water and soil, and their bioavailability associated with AMR selection; and (8) risk assessments of AMR and related pathogens (104). These data are lacking. Limitations for acquiring these data includes some soil bacteria are difficult to culture and acquiring accurate data from flowing water is difficult as it is inherently dynamic and diluting (18).

As much of the world's population, including Canadians (105), obtain drinking water from excreta-impacted waters that likely contain AMR bacteria, there is an urgent need to better understand what concentration limits should be considered to better manage potential mass-inoculation of people with resistance genes (106). However, the only antimicrobial concentration limits under consideration relate to wastewaters (107). Not surprisingly, there are AMR genes in Canadian urban sewage (90, 108). Furthermore, the prevalence of AMR genes in treated effluent leaving water treatment facilities in the Canadian prairies provides evidence for human influence on local environmental reservoirs of resistance (108).

Without considering all drivers and pathways of AMR introduction into the environment, any strategy to reduce resistance across all sectors risks failure (18). To mitigate development and transmission of AMR bacteria, the environment must be considered an important reservoir and policy changes must encompass agricultural and environmental best practices along with those in human and veterinary medicine. Currently, a key knowledge gap in AMR transmission prevention is limited understanding of quantitative risks to identify the relative importance of various AMR development and transmission incursion pathways (Table 1).

## Discussion

There are numerous knowledge gaps in understanding AMR in Canada. AMR is complex, with many contributing factors, numerous antimicrobial end users, countless microbial interactions, and varying policies and regulatory capacities around the world. Substantial progress will require much investment in AMR research, surveillance, and capacity building, coupled with effective policies at national, provincial/territorial and local levels.

**Figure 2:**
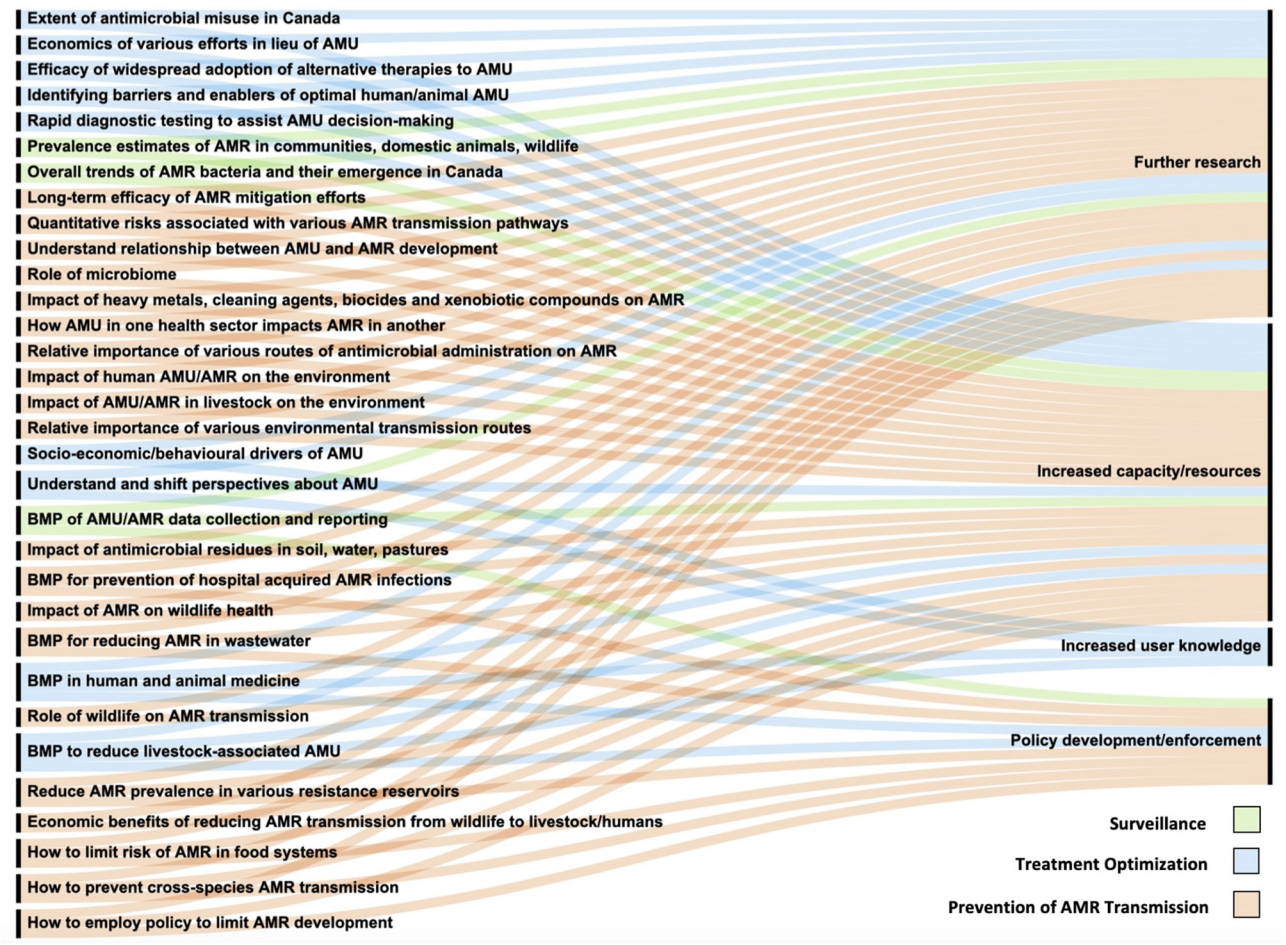
Knowledge gaps and their requirements to be addressed as organized into four categories. BMPs, Best management practices; AMU, Antimicrobial use; AMR, Antimicrobial resistance.

Various knowledge gap assessments (16, 109) and action plans have been developed regarding AMR, including the WHO Global Action Plan (3) and the Federal action plan on antimicrobial resistance and use in Canada (99). Most requirements detailed in this review (Figure 2), developed for the three key areas (treatment optimization, surveillance, and transmission), are logical continuations of described action plans with focus on the Canadian context. Moreover, the One Health approach used in this review identified additional novel gaps for Canada. Prioritization of required investments and activities must be considered with further cooperation and coordination among existing initiatives. Required efforts to address described knowledge gaps are described in Figure 2 and separated into categories: (1) further research, (2) increased capacity/resources, (3) increased prescriber/end-user knowledge, and (4) policy development/enforcement. Each knowledge gap has been assigned one or more categories as required changes for the gap to be addressed.

Research and increased capacity/resources are required to fully address all knowledge gaps. Required research is similar across the human, animal, and environmental sectors (108) and will enable development of specific recommendations, to ensure they are effective and practical. Otherwise, general uptake and AMR mitigation may be limited. Furthermore, understanding of environmental components is severely lacking, and requires immediate research and capacity building to ensure AMR mitigation efforts are effective. In contrast, increasing end-user knowledge regrading appropriate AMU and challenging clinical visit expectations in both human and animal medicine is a shift in focus from research to increasing end-user knowledge. However, this must include further understanding of drivers and barriers of AMS to increase recommendation uptake to facilitate sustained behavior change. Research surrounding this will focus on bridging the gap between knowledge and practice.

Increased capacity/resources should accompany further research, as increased funding, research activity design/development, laboratory capacity, data analysis and publishing/knowledge transfer are required for research to be effective and to inform decisions in policy and influence end-user decision-making. However, capacity and financial support for required research may be inadequate. For example, Canada's research and development support for new antimicrobials is severely lacking (110). Therefore, without an incentivized market for new antimicrobial development, progress fostering required research is unlikely.

An emphasis on “further research” and “increased capacity/resources” does not imply that other requirements are less important. Rather, it highlights a lack of required knowledge and understanding to develop effective policy/recommendations. The lack of best management practices for AMU and AMS programs, serve as an example.

Reductions in prophylactic AMU were not considered possible but became feasible with better farm management and biosecurity. However, not all food animal industries are equally ready to implement management practices that reduce AMU. Research into best management practices is required for sustained AMU reduction that does not negatively impact animal health and welfare. However, this will require substantial investment in long-term research to establish a causal link between AMU reductions and AMR implications which progresses into practical policy development and enforcement. The Canadian poultry industry had some success with a voluntary AMS program (51, 54). After banning 3rd generation cephalosporins in 2014, *Salmonella* isolates from sick people, and *Salmonella* and *E. coli* isolates from chickens at slaughter and retail meat had lower 3rd generation cephalosporin resistance (51). However, in 2018, despite no reported 3rd generation cephalosporin use, there was a slight increase in healthy chickens with *Salmonella* resistant to these products (51).

Additionally, further collaborative surveillance programs are required to assess the degree of human impact on various ecosystems and determine best relative mitigation efforts. Despite being previously identified as an important gap (30), substantial progress has not occurred. This should also include research regarding impacts of co-selection of AMR by common cleaning agents in animal production facilities and human hospitals (18). This should be coupled with research into transmission of AMR bacteria between species, AMR contamination pathways of groundwater, including discharge of antimicrobial residues in sewage and disposal of antimicrobial waste (i.e., unused or expired drugs, packaging with residual drug).

Although surveillance is essential to understanding AMR and AMU trends, generalization of their results across bacteria or antimicrobials can falsely inform mitigation efforts. A substantial aid in development of AMS best practices and prioritization efforts will be quantitative risk-based assessments of AMR development and the relationship among human, animal, and environmental sectors. With risk-based models, policymakers can be better informed to use available resources to target areas of high importance. Coupled with the social and behavioral research components, this will promote improvements on a national level.

Prioritization of required activities is needed to focus efforts and available resources; however, without answers to some of these knowledge gaps, it is difficult to identify the most pressing areas to act (i.e., restrict AMU, prescribing best practices, environmental containment, etc.). Although, in general, AMR understanding and mitigation attempts should follow the following roadmap: (1) detailed understanding of missing data required to inform realistic policies and best management practices, (2) implementation and ongoing evaluation of policies and recommendations at the community, provincial/territorial, and national levels, (3) sustainable implementation with ongoing evaluation to account for new innovations and AMU/AMR surveillance findings (16). Additionally, to lay the groundwork for changes in antimicrobial prescribing and use, must be accompanied by public educational campaigns to improve general understanding of the risks associated with AMR and unnecessary AMU, but also to increase social responsibility. In summary, AMS cannot be considered as separate actions in human, animal, and environmental sectors. Bacteria that develop resistance in one sector have potential to extend to other sectors; therefore, immediate, and concerted action across all sectors is necessary. This can be done by taking a One Health approach to optimize global health and well-being.

Prudent AMU can still allow development of AMR; however, rigorous management can limit the risks (1). The victory over AMR is never final; rather there is an ongoing battle that requires constant surveillance and AMS practice. With continued surveillance and research into the described knowledge gaps, a more thorough understanding of the complex relationships between microbial communities can be obtained. Additionally, behaviors of antimicrobial prescribers, consumers, and distributors require further investigation, as they are key to appropriate AMS measures. If this is combined with collaborative and sustained mitigation efforts across all sectors, we can preserve effective treatment options for bacterial infections throughout the One Health continuum in the future.

## Author Contributions

This manuscript has been prepared by KDMcC, with supervision from HB and RMA. EdJ, JI, DN, JK, JC, MG, TM, KO, IL, LJ, GP, H-JW, KMcC, ML, JR, LH, AH, NA, SC, GT, AB, ER-M, EG, and SO contributed significantly to the intellectual content related to their respective fields. All authors contributed to manuscript revision, as well as read, and approved the submitted version.

## Funding

This research was part of the Antimicrobial Resistance–One Health Consortium, which is predominantly financed by the Ministry of Jobs, Economy and Innovation, Alberta, Canada. KDMcC was supported by Canadian Dairy Commission Workplace Development Initiative Scholarship, NSERC CREATE in Milk Quality Program Scholarship and the NSERC Alexander Graham Bell Canada Graduate Scholarship.

## Conflict of Interest

JC has received peer-reviewed research grants from the Canadian Institutes for Health Research (CIHR) on development of a rapid assay for detection of methicillin-resistant *Staphylococcus aureus* and Alberta Innovates Health Solutions on use of probiotics as primary prophylaxis for *Clostridium difficile* colitis. JC has also received financial support from Pfizer for the STRIVE *S. aureus* vaccine randomized clinical trial in vertebral spinal surgery with instrumentation and CIHR for creation of an AMS app to improve antimicrobial prescribing. The remaining authors declare that the research was conducted in the absence of any commercial or financial relationships that could be construed as a potential conflict of interest.

## Publisher's Note

All claims expressed in this article are solely those of the authors and do not necessarily represent those of their affiliated organizations, or those of the publisher, the editors and the reviewers. Any product that may be evaluated in this article, or claim that may be made by its manufacturer, is not guaranteed or endorsed by the publisher.
